# Initial uptake, time to treatment, and real-world effectiveness of all-oral direct-acting antivirals for hepatitis C virus infection in the United States: A retrospective cohort analysis

**DOI:** 10.1371/journal.pone.0218759

**Published:** 2019-08-22

**Authors:** Paul Y. Kwo, Amy Puenpatom, Zuoyi Zhang, Siu L. Hui, Andrea A. Kelley, David Muschi

**Affiliations:** 1 Department of Medicine – Gastroenterology and Hepatology, Stanford University School of Medicine, Palo Alto, California, United States of America; 2 Center for Observational and Real-World Evidence (CORE), Merck & Co., Inc., Kenilworth, New Jersey, United States of America; 3 Department of Medicine, Regenstrief Institute, Indianapolis, Indiana, United States of America; 4 Department of Biostatistics, Regenstrief Institute, Indianapolis, Indiana, United States of America; 5 Data Core Services, Regenstrief Institute, Indianapolis, Indiana, United States of America; 6 Department of Biostatistics, Indiana University School of Medicine, Indianapolis, United States of America; Nihon University School of Medicine, JAPAN

## Abstract

**Background:**

Data on initiation and utilization of direct-acting antiviral therapies for hepatitis C virus infection in the United States are limited. This study evaluated treatment initiation, time to treatment, and real-world effectiveness of direct-acting antiviral therapy in individuals with hepatitis C virus infection treated during the first 2 years of availability of all-oral direct-acting antiviral therapies.

**Methods:**

A retrospective cohort analysis was undertaken using electronic medical records and chart review abstraction of hepatitis C virus-infected individuals aged >18 years diagnosed with chronic hepatitis C virus infection between January 1, 2014, and December 31, 2015 from the Indiana University Health database.

**Results:**

Eight hundred thirty people initiated direct-acting antiviral therapy during the 2-year observation window. The estimated incidence of treatment initiation was 8.8%±0.34% at the end of year 1 and 15.0%±0.5% at the end of year 2. Median time to initiating therapy was 300 days. Using a Cox regression analysis, positive predictors of treatment initiation included age (hazard ratio, 1.008), prior hepatitis C virus treatment (1.74), cirrhosis (2.64), and history of liver transplant (1.5). History of drug abuse (0.43), high baseline alanine aminotransferase levels (0.79), hepatitis B virus infection (0.41), and self-pay (0.39) were negatively associated with treatment initiation. In the evaluable population (n = 423), 83.9% (95% confidence interval, 80.1–87.3%) of people achieved sustained virologic response.

**Conclusion:**

In the early years of the direct-acting antiviral era, <10% of people diagnosed with chronic hepatitis C virus infection received direct-acting antiviral treatment; median time to treatment initiation was 300 days. Future analyses should evaluate time to treatment initiation among those with less advanced fibrosis.

## Introduction

Hepatitis C virus (HCV) infection is the most common blood-borne infection in United States, with approximately 2.2 to 3.8 million persons infected nationwide [1]. Left untreated, HCV infection can cause cirrhosis or hepatocellular carcinoma, and may lead to liver transplant or death [2]. Before 2011, interferon-based therapies were the main treatment for HCV infection, yielding sustained virologic response (SVR, or cure) rates of approximately 45% to 50% [3]. The introduction of first-generation direct-antiviral agents (DAA), administered with pegylated interferon and ribavirin, improved SVR rates to approximately 50% to 75% in treatment-naive individuals with genotype (GT) 1 infection [4–6]. However, the improvements in SVR attained with first-generation agents were associated with an increased frequency of adverse events. In 2013, simeprevir and sofosbuvir were approved for the treatment of HCV infection [7]. Although not approved as a combination regimen, the off-label use of these agents led to the first all-oral DAA therapy for HCV GT1 infection. In addition, sofosbuvir plus ribavirin was approved for the treatment of GT2 and GT3 infection [8]. Subsequently, ledipasvir/sofosbuvir and ombitasvir, paritaprevir, and dasabuvir were approved in the United States and Europe. These agents offered short-duration, interferon-free treatments that could be administered with or without ribavirin and achieved SVR rates of >90% [9–14].

Additional barriers to the real-world treatment of HCV infection exist that are often controlled for in clinical trials. Real-world treatment of HCV infection may be complicated by comorbidities and concomitant medications, and economic considerations can prevent the successful initiation or completion of DAA-based therapy [15–18]. To date, data are limited concerning the proportion of HCV-infected individuals who are eligible for DAA treatment or who have initiated treatment, and few reports have evaluated the time to treatment initiation following diagnosis. The objectives of this study were to evaluate treatment initiation rates, time to treatment, and predictors of initiating treatment in individuals receiving all-oral DAA therapy in a real-world setting. Real-world effectiveness of all-oral DAA regimens and early discontinuation rates were also assessed.

## Methods

This was a retrospective cohort analysis using electronic medical records (EMRs) and chart review abstraction of HCV-infected individuals from the Indiana University health database, which included data from 48,000 persons in 2015. The study was conducted in accordance with the Declaration of Helsinki and Good Clinical Practice guidelines, and the protocol was approved by the Indiana University Office of Research Compliance institutional review board. Data regarding prior HCV treatment and comorbidities were captured through the Indiana Network for Patient Care, a health information exchange containing data from multiple Indiana healthcare institutions. Manual chart reviews were performed to validate HCV genotype, treatment start and end dates, and clinical characteristics including HCV RNA viral load, aspartate aminotransferase (AST) and alanine aminotransferase (ALT) levels, and platelet counts. A waiver of the informed consent process was approved by the appropriate institutional review board.

### Study population

Individuals aged >18 years with a documented ICD-9 and/or ICD-10 code (International Classification of Diseases, 9^th^ or 10^th^ edition, Clinical Modification) of non-acute HCV infection and a documented encounter between January 1, 2014, and December 31, 2015 (the cohort identification period) were included. All participants with at least one record of an all-oral DAA prescription filled during the cohort identification period were included in the DAA-treated cohort. End-of-treatment for participants enrolled during the cohort identification period could extend to March 31, 2016. DAA regimens in this study included sofosbuvir/ledipasvir ± ribavirin, paritaprevir/ritonavir/ombitasvir/dasabuvir (PrOD) ± ribavirin, sofosbuvir/simeprevir ± ribavirin, and sofosbuvir + ribavirin. Participants were required to have ≥6 months of historical records prior to the treatment index date, which was defined as the time at which an individual first received all-oral DAA treatment during the study period. Individuals receiving concurrent interferon therapy were excluded from the DAA-treated cohort.

HCV-infected individuals with no record of all-oral DAA treatment were included in the HCV untreated cohort.

### Study measures

Baseline characteristics, prior HCV treatment, and HCV genotype were recorded. HCV genotype, fibrosis level, AST:platelet ratio index (APRI), intended treatment duration, and treatment completion duration were extracted from medical chart reviews. Fibrosis level was based on the APRI and Fibrosis 4 (FIB-4) indices. The FIB-4 score was calculated based on variables recorded before the DAA index date, using the following formula:

$$\text{FIB}-4=\text{age}[\text{years}]\times \text{AST}[\text{IU}/\text{L}]/\text{platelet}\;\text{count}[\times 10^{9}/\text{L}]\times (\text{AL}{\text{T}}^{1/2}[\text{IU}/\text{L}])$$

APRI score was calculated via the following formula:

$$\text{APRI}=\text{aminotransferase}/\text{normality}\;\text{upper}\;\text{limit}/\text{platelet}[10^{9}/\text{L}]\times 100$$

Cirrhosis was defined as a FIB-4 score of >3.5. Co-morbidities such as hepatitis B virus infection, HIV, and history of kidney or liver transplant were defined by the presence of at least one ICD-9 CM or ICD-10 CM code during the baseline period of 1 year prior to and including the index date.

### Study outcomes

Treatment initiation rates and time to treatment were calculated using the time from the first clinical encounter of HCV diagnosis to the first medication order of a DAA agent. Early treatment discontinuation was defined as an observed treatment duration minus treatment gap that was shorter than intended treatment duration by 14 days. SVR12 was defined as undetectable HCV RNA 12 weeks after the end of treatment, and end of treatment was calculated as the last date covered by prescription using medication dispensed information and number of days for which medication was supplied. SVR12 was calculated for the evaluable population (all participants with an available treatment outcome who completed the planned regimen or discontinued early) and the per-protocol population (all participants with an available treatment outcome who completed treatment).

### Statistical analysis

Descriptive statistics and univariate analysis were used to describe the distribution of demographics and clinical and laboratory characteristics. Time to treatment was used to estimate the cumulative percentage of eligible HCV-infected individuals receiving DAA prescriptions. Individuals with no DAA prescription were assumed to have not received any treatment at Indiana University until the last encounter in the EMR, at which point the observed time to treatment was censored. Kaplan–Meier analysis was used to estimate the percentage of participants receiving prescriptions at any time from the first clinical encounter.

Univariate analysis was used to identify factors significantly associated with DAA prescription using *t* tests and chi-square tests. Cox regression analysis was used to predict factors associated with time to initiation of therapy. SAS (version 9.4, SAS Institute Inc., Cary, NC, USA) and Stata version 11 (Stata Corp., College Station, TX, USA) were utilized to conduct analyses.

## Results

A total of 8,611 individuals with a diagnosis of chronic HCV, or a pharmacy order for a DAA agent between January 1, 2014, and December 31, 2015, were identified. After applying inclusion/exclusion criteria, 8,407 individuals with chronic HCV infection were included, of whom 830 initiated DAA therapy and 7,577 did not, resulting in a treatment initiation rate of 9.9%.

Individuals in the DAA-treated cohort were older than those in the untreated cohort (median age, 57 years versus 52 years, p<0.0001), and there were fewer women in the treated versus untreated cohort (39.5% versus 45.3%, p = 0.0014) (Table 1). Most were white (DAA-treated cohort, 79.9%; untreated cohort, 79.2%) and had HCV GT1a infection (56.1% vs. 60.8%). Compared with the untreated cohort, people in the treated cohort were more likely to have cirrhosis (43.5% vs. 15.4%, p<0.0001), a higher severity of Charlson comorbidity index (p<0.0001), and a history of kidney (1.7% vs. 0.7%, p = 0.0024) or liver transplant (14.1% vs. 4.4%, p<0.0001). Fewer people in the DAA-treated cohort had a history of depression (7.6% vs. 11.7%, p<0.0003) or drug abuse (5.8% vs. 12.7%, p<0.0001) compared with the untreated cohort. A higher percentage of individuals in the DAA-treated cohort had previously received interferon-based therapies compared with the untreated cohort (25.5% vs. 7.1%, p<0.0001). Proton pump inhibitor use was lower in the treated compared with the untreated cohort (36.9% vs. 42.4%, p = 0.0022).

**Table 1:** Participant characteristics and demographics.

Characteristic	DAA-Treated Cohort (N = 830)	Untreated Cohort (N = 7,577)	p value
Age, mean (SD)	56.9 (9.3)	52.1 (13.3)	<0.0001
Sex, n (%)			
Female	328 (39.5)	3,434 (45.3)	0.0014
Race, *n* (%)			
Black	152 (18.6)	1,408 (18.9)	0.7948
White	654 (79.9)	5,917 (79.2)	
Other	13 (1.6)	142 (1.9)	
Missing, n	11	110	
Ethnicity, n (%)			
Hispanic or Latino	18 (2.2)	132 (1.8)	0.3915
Missing	13	164	
Insurance, n (%)			
Government	50 (6)	684 (9.1)	<0.0001
Medicaid	131 (15.8)	1,639 (21.9)	
Medicare	323 (39)	2,318 (31)	
Private	301 (36.3)	2,000 (26.7)	
Self-Pay	24 (2.9)	823 (11)	
Workman’s Comp	0	14 (0.2)	
Missing	1	99	
ALT level, mean (SD), IU/L	54.1 (51.9)	45 (92.7)	0.0003
Missing, n	137	4128	
Anxiety, n (%)			
Yes	55 (6.6)	766 (10.1)	0.0013
APRI, mean (SD)	1.4 (1.5)	1.1 (2.1)	<0.0001
Missing, n	158	4454	
AST level, mean (SD), IU/L	55.9 (40.7)	47.3 (92.5)	0.0001
Missing, n	140	4154	
Charlson Comorbidity Index, n (%)			
0	294 (35.4)	5,943 (78.4)	<0.0001
1	272 (32.8)	616 (8.1)	
2	59 (7.1)	265 (3.5)	
≥3	205 (24.7)	753 (9.9)	
Cirrhosis, n (%)			
Yes	361 (43.5)	1,165 (15.4)	<0.0001
CKD, n (%)			
Yes	53 (6.4)	493 (6.5)	0.8932
Decompensated cirrhosis, n (%)			
Yes	200 (24.1)	820 (10.8)	<0.0001
Depression, n (%)			
Yes	63 (7.6)	889 (11.7)	0.0003
Diabetes, n (%)			
Yes	105 (12.7)	977 (12.9)	0.8422
Drug abuse, n (%)			
Yes	48 (5.8)	965 (12.7)	<0.0001
FIB-4, n (%)			
>3.5	313 (46.6)	909 (29.3)	<0.0001
Missing, n	158	4474	
Fibrosis stage, n (%)			
F0	51 (6.6)	33 (14.7)	<0.0001
F1	118 (15.3)	63 (28.1)	
F2	87 (11.3)	36 (16.1)	
F3	82 (10.6)	15 (6.7)	
F4	433 (56.2)	77 (34.4)	
Missing	59	7353	
Genotype, n (%)			
GT1	51 (6.4)	35 (1.4)	<0.0001
GT1a	445 (56.1)	1,539 (60.8)	
GT1b	117 (14.8)	407 (16.1)	
GT2	93 (11.7)	257 (10.2)	
Other	87 (11)	294 (11.6)	
Missing	37	5045	
HBV infection, n (%)			
Yes	9 (1.1)	157 (2.1)	0.0522
HIV, n (%)			
Yes	13 (1.6)	136 (1.8)	0.6355
History of kidney transplant, n (%)			
Yes	14 (1.7)	53 (0.7)	0.0024
History of liver transplant, n (%)			
Yes	117 (14.1)	336 (4.4)	<0.0001
Neutropenia, n (%)			
Yes	3 (0.4)	36 (0.5)	0.6473
Platelet count ≥100,000/μL, n (%)	468 (67.3)	2,933 (79.4)	<0.0001
Missing, n	135	3,882	
Previous treatment (interferon, pegylated interferon, ribavirin, first-generation DAAs), n (%)			
Yes	212 (25.5)	536 (7.1)	<0.0001
Thrombocytopenia, n (%)			
Yes	19 (2.3)	99 (1.3)	0.0223
ß-blocker use, n (%)			
Yes	318 (38.3)	3,377 (44.6)	0.0006
Proton pump inhibitor use, n (%)			
Yes	306 (36.9)	3,211 (42.4)	0.0022

ALT, alanine aminotransferase; APRI, aspartate aminotransferase to platelet ratio index; AST, aspartate aminotransferase; CKD, chronic kidney disease; DAA, direct-acting antiviral; FIB-4, Fibrosis 4; HBV, hepatitis B virus; HIV, human immunodeficiency virus.

### Time to treatment initiation and factors associated with treatment initiation

**Fig 1:**
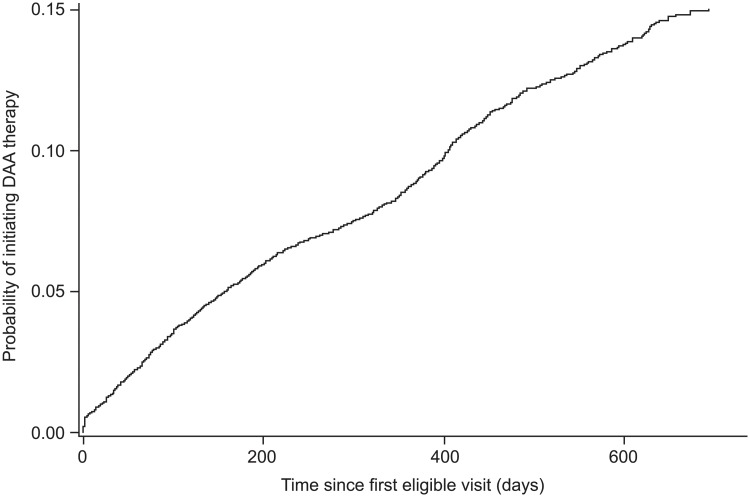
Kaplan–Meier curve: Time to treatment initiation.

At year 1, the estimated incidence of treatment initiation was 8.80% ± 0.34%, and at year 2, this increased to 15% ± 0.5%. The median time to DAA initiation was 300 days among those who received treatment within the 2-year observation window (Fig 1), ranging from 25 to 675 days with a distribution as shown in S1 Fig. Sofosbuvir/ledipasvir ± ribavirin was the most commonly initiated treatment, comprising 382/830 (46%) of all treatments started, followed by sofosbuvir/ribavirin (25.7%) and sofosbuvir/simeprevir ± ribavirin (22.5%) (Table 2).

**Table 2:** Distribution of direct-acting antiviral treatments and early treatment discontinuation by regimen.

	Treatment Regimens						
Treatment initiation/early discontinuation	Sofosbuvir/ledipasvir	Sofosbuvir/ledipasvir + Ribavirin	Sofosbuvir + Ribavirin	Sofosbuvir/simeprevir ± Ribavirin	PrOD ± Ribavirin	Other DAA Agents	Total
DAA therapy initiation, n (% of total treatment initiations)	261 (31.45)	121 (14.58)	213 (25.66)	187 (22.53)	20 (2.41)	28 (3.37)	830^a^ (100.0)
Early discontinuation, n (% of total early treatment discontinuations)	6 (2.30)	3 (2.48)	14 (6.57)	8 (4.28)	2 (10.00)	2 (7.14)	35 (4.22)

DAA, direct-acting antiviral; PrOD, paritaprevir/ritonavir/ombitasvir/dasabuvir.

^a^9.87% of 8,407 in total eligible cohort.

Positive predictors of treatment initiation included age (hazard ratio [HR] 1.01), treatment-experienced (HR 1.74), cirrhosis (HR 2.63), and history of liver transplant (HR 1.5). History of drug abuse (HR 0.43), elevated baseline ALT (HR 0.79), a history of hepatitis B virus infection (HR 0.41), and self-pay for medical care (HR 0.39) were negatively associated with treatment initiation (Table 3).

**Table 3:** Predictors of initiation of direct-acting antiviral treatment by Cox regression analysis.

Characteristic	Coefficient	Hazard Ratio	HR Lower CL	HR Upper CL	*P* value
Age	0.01	1.01	1.00	1.02	0.042
Female	−0.32	0.73	0.51	1.03	0.074
Insurance					
Medicaid	−0.44	0.65	0.47	0.90	0.009
Medicare	−0.32	0.72	0.53	0.98	0.039
Private	−0.07	0.94	0.69	1.27	0.663
Self-Pay	−0.93	0.39	0.24	0.64	<0.001
Workman’s Comp	−10.06	0	0	1.35+143	0.954
Unknown insurance type	−2.42	0.09	0.01	0.65	0.017
Elevated ALT level^a^	−0.24	0.79	0.54	1.15	0.213
Cirrhosis	0.97	2.64	2.24	3.10	<0.0001
History of drug abuse	−0.85	0.43	0.32	0.58	<0.0001
HBV infection	−0.88	0.41	0.21	0.80	0.009
History of liver transplant	0.40	1.50	1.22	1.83	<0.001
Elevated platelet count^b^	0.15	1.16	0.97	1.39	0.112
Missing platelet data	−0.38	0.69	0.50	0.94	0.018
Treatment-experienced	0.55	1.74	1.47	2.05	<0.0001

ALT, alanine aminotransferase; CL, confidence limit; HBV, hepatitis B virus; HR, hazard ratio.

^a^Elevated ALT level: ALT ≥33 IU/L for males; ≥25 IU/L for females.

^b^Elevated platelet count: ≥100,000/μL; low: <100,000/μL.

Positive references were male, treatment naive, and had government insurance, low ALT levels, no cirrhosis, no history of drug abuse, no HBV infection, no history of liver transplant, low platelet count.

Manual chart review of a random sample from the untreated cohort (n = 287) suggests that the most common reasons for lack of initiation of therapy were absence of patient follow-up (33.7%) and insurance declined or other cost barrier (28.1%). Physician decision not to treat accounted for 16% of non-initiation cases. The reasons for not initiating DAA therapy are listed in S1 Table.

Clinical characteristics and demographics of the treated cohort stratified by DAA regimen are reported in S2 Table. Ribavirin was added to sofosbuvir/ledipasvir most commonly for individuals with more advanced fibrosis, including compensated and decompensated cirrhosis, those with prior liver transplant, and those previously treated with an interferon-based therapy.

### Overall SVR rates and SVR by subgroup

In the evaluable population cohort (n = 423), 83.9% (95% confidence interval [CI] 81–87%) of participants achieved SVR (Table 4). Excluding those who did not complete treatment, SVR in the per-protocol population was 84.7% (343/405; 95% CI 81–88%) regardless of genotype. For the evaluable population, SVR rates were 85.0% (153/180) in women, 83.1% (202/243) in men, 82.5% (104/126) in treatment-experienced participants, 78.2% (154/197) in cirrhotic participants, and 88.7% (220/248) in those with platelet count ≥100,000/μL. SVR rates were 84.7% (261/308) in participants treated for 12 weeks versus 82.9% (68/82) in those treated for 24 weeks, and 83.3% (15/18) in those treated for 8 weeks in the evaluable population (Table 4).

**Table 4:** Sustained virologic response rates (95% confidence intervals) in participants with HCV GT1 infection who initiated direct-acting antiviral treatment^a^.

	Evaluable Population		Per-Protocol Population	
	No. of Participants Achieving SVR, % (95% CI)	n	No. of Participants Achieving SVR, % (95% CI)	n
All participants	355 83.92 (81–87)	423	343 84.69 (81–88)	405
8-week treatment	15 83.33 (59–96)	18	14 82.35 (57–96)	17
12-week treatment	261 84.74 (80–89)	308	260 85.25 (81–89)	305
24-week treatment	68 82.93 (73–90)	82	68 82.93 (73–90)	82
SVR by subgroup				
Female	153 85.00 (79–90)	180	148 84.06 (79–90)	174
Male	202 83.13 (78–88)	243	195 84.42 (79–89)	231
Cirrhosis	154 78.17 (72–84)	197	149 78.42 (72–84)	190
No cirrhosis	201 88.94 (84–93)	226	194 90.23 (85–94)	215
Black	82 92.13 (84–97)	89	79 94.05 (87–98)	84
Non-black	273 81.74 (77–86)	334	264 82.24 (78–86)	321
Treatment-experienced	104 82.54 (75–89)	126	100 82.64 (75–89)	121
Treatment-naive	251 84.51 (80–88)	297	243 85.56 (81–89)	284
Platelet count ≥100,000/μL	220 88.71 (84–92)	248	211 89.79 (85–93)	235
Platelet count <100,000/μL	86 74.78 (66–82)	115	83 74.45 (66–83)	110

CI, confidence interval; DAA, direct-acting antiviral; GT, genotype; HCV, hepatitis C virus; SVR, sustained virologic response.

^a^SVR was estimated among participants with available SVR measurements.

The SVR rates by DAA treatment are listed in S3 Table. Among participants with GT1 infection, the overall unadjusted SVR rate was 83.9%. SVR was achieved by 92.2% (n = 53/166) of participants receiving sofosbuvir/ledipasvir, with slightly lower SVR rates in those with cirrhosis compared with no cirrhosis (88.9% vs. 94.2%). Of the 65 individuals who received sofosbuvir/ledipasvir + ribavirin, 84.6% achieved SVR, with a slightly lower rate of SVR in cirrhotic participants. The SVR rate among the 132 participants who received sofosbuvir/simeprevir ± ribavirin was 79.6%, with lower rates in those with cirrhosis (73.4%) and treatment-experienced individuals (79.3%). Ten individuals initiated treatment with PrOD ± ribavirin, with an SVR rate of 90% (9/10). Forty-six individuals initiated therapy with sofosbuvir + ribavirin, with an overall SVR rate of 65.2%, and 59.3% in those with cirrhosis.

The treatment discontinuation rate was 4.2%, ranging from 2.3% in people receiving sofosbuvir/ledipasvir to 6.6% in those receiving sofosbuvir/ribavirin and 10% in those receiving PrOD ± ribavirin.

## Discussion

All-oral DAA therapy has revolutionized the treatment of chronic HCV infection. Before the availability of DAA agents, SVR rates of up to 75% were observed in HCV GT1-infected individuals; however, side effects with interferon-based treatments were often severe, and many individuals were not eligible for interferon therapy. SVR rates of >95% have been reported with all-oral DAA combinations in individuals with GT1 infection, as confirmed in multiple observational cohort studies [19–21]. However, the benefits of highly efficacious therapies may be limited if these treatments cannot be accessed by those who require them. To date, data regarding the frequency of treatment initiation in the era of DAA therapy are limited. This study therefore serves as a reference for newer DAA therapies with improved access to care.

The demographics of this study population mirror those of other HCV infection cohorts reported in registration trials, with most participants being white males [2, 8, 9, 11]. The overall uptake of therapy among participants eligible for treatment during the first 2 years of the DAA era was low at 9.9%, and was similar to that previously reported with interferon-based treatments [22]. We observed an increase in the incidence of treatment initiation during the second year of the study; however, a median time to initiation of therapy of 300 days suggests that substantial improvements in overcoming barriers to treatment are still required. Insurance type was a significant predictor of treatment initiation, with the highest rates seen in those with Medicare and private insurance. Disease severity was also a predictor of initiation of therapy, with higher rates in those with clinical features suggesting advanced liver disease, including compensated and decompensated cirrhosis, greater Charlson comorbidity scores, and a history of liver transplant. It is likely that the higher rates of treatment initiation in these groups can be explained partially by their requirement for continuous medical care. Prior treatment was also correlated with initiation of treatment, whereas a history of drug abuse was negatively correlated with treatment initiation. The characteristics of our treatment cohort mirror the population initially prioritized for treatment in guidelines and by private and public insurers. Future studies should examine fibrosis status among those initiating therapy to determine if those with less severe disease and fewer comorbidities are also starting treatment. Current guidelines emphasize that all individuals with HCV infection should receive DAA therapy if they comply with the intended treatment [23]. More recent data suggest that individuals with depression, illicit substance use, and other psychiatric disorders can be successfully treated, with SVR rates similar to those without these comorbidities. It is also expected that future studies will demonstrate that a history of drug abuse does not negatively correlate with initiation of therapy [24, 25]. In our study, the highest rates of treatment initiation were 39% and 36.3% in people with Medicare and private insurance, respectively, compared with only 15.9% of those with Medicaid. However, in several states treatment restrictions are gradually being reduced, which, given the sizeable population of HCV-infected individuals with Medicaid as their primary insurance, should improve DAA therapy initiation rates.

The most common intended treatment duration was 12 weeks with sofosbuvir/ledipasvir, with an overall SVR of 92.2% (n = 153/166). In people receiving sofosbuvir/ledipasvir, SVR rates were lower in cirrhotic than in non-cirrhotic individuals (88.9% vs. 94.2%). Those who received sofosbuvir/ledipasvir + ribavirin had an overall response rate of 84.6%, with no difference between treatment-naive and treatment-experienced individuals (84.2% vs. 85.2%). Other reports confirm that the real-world effectiveness of DAA agents varies by HCV population. HCV-TARGET is a prospective, longitudinal, observational study of patients with chronic HCV infection at academic and community centers from the United States, Canada, Germany, and Israel. This study has reported SVR rates of 84% in people with HCV GT1 infection receiving sofosbuvir/simeprevir for up to 16 weeks [19]; 94% in people with HCV GT1 infection receiving sofosbuvir/ledipasvir for 12 or 24 weeks [26]; and 93% in individuals with HCV GT1 infection receiving sofosbuvir/ledipasvir for 8 to 16 weeks [20]. In addition, studies from the Veterans Affairs population in the United States and a large US commercially insured population evaluating patients with HCV GT1 infection confirm SVR rates of 90% to 94% in individuals receiving sofosbuvir/ledipasvir [27–30].

An SVR rate of 79.5% was noted in those who received sofosbuvir/simeprevir with or without ribavirin. The combination of sofosbuvir/simeprevir is approved for the treatment of GT1-infected individuals [31]; however, in 2014 this was an off-label combination that represented the most efficacious regimen available for people with GT1 infection. In other real-world settings, rates of SVR12 in people receiving sofosbuvir/simeprevir were 64% in those with GT1a infection and 84% in those with GT1b infection [32]. SVR rates of 72% in those with GT2 infection receiving sofosbuvir/ribavirin and 35% in those with GT3 infection receiving sofosbuvir/ribavirin are also reported [32].

Early discontinuation rates were low (4.2%), ranging from 2.3% in participants receiving sofosbuvir/ledipasvir without ribavirin to 10% in those receiving PrOD ± ribavirin. These discontinuation rates are substantially lower than those reported with interferon-based therapy, likely owing to the dramatically different tolerability profiles between DAA and interferon-based treatments [3]. It is also apparent that discontinuation rates in the present study were higher among participants receiving a ribavirin-containing regimen, possibly because of the well-described tolerability profile of ribavirin. Welzel et al. also reported discontinuation rates of 6.7% in people receiving sofosbuvir/ribavirin in a real-world setting; however, other studies indicate that discontinuation rates with DAA therapies are similar (approximately 1% to 3%) regardless of ribavirin use [19, 20].

This study had several limitations. The source of the database was a large center in Indiana; thus, the findings may not be generalizable to the general US population. The study period included the first 2 years that all-oral DAA therapies were available; since this time, both guidelines and insurers have broadened the criteria for treatment eligibility, with the present American Association for the Study of Liver Diseases guidelines stating that all people with HCV infection without a limited lifespan and who can comply with treatment should be considered for therapy. Since these data were collected, there have been substantial changes in the use of DAA therapies for the treatment of HCV infection; these data are therefore unlikely to accurately represent the current utilization of DAA treatments. The use of EMRs may limit the granularity or the completeness of our data. We therefore implemented a manual chart review to minimize the impact of missing values and to validate laboratory results.

## Conclusion

Data from the present study provide insight into initiation of DAA therapy during the early years of the availability of all-oral regimens. We found that treatment was initiated in 9.9% of HCV-infected individuals and time to initiation was 300 days. Expanding treatment eligibility criteria and reducing economic barriers should increase rates of treatment initiation and reduce time to treatment. Future studies, based on more recent treatment periods, are likely to demonstrate improved access to treatment and broadened treatment eligibility including individuals with less advanced disease.

## Supporting information

S1 Table Reasons for not initiating treatment (sample of untreated cohort with mention of ‘DAA’ in patient notes). (DOCX)

S2 Table Baseline characteristics and demographics of the direct-acting antiviral-treated cohorts stratified by regimen (N = 830). (DOCX)

S3 Table Sustained virologic response rates by direct-acting antiviral treatment regimen in participants with HCV GT1–infection (evaluable population). (DOCX)

S1 Fig Histogram of time to treatment initiation. (DOCX)
