# Supplementary material for: Initial uptake, time to treatment, and real-world effectiveness of all-oral direct-acting antivirals for hepatitis C virus infection in the United States: A retrospective cohort analysis
Source: PLoS One. 2019 Aug 22;14(8):e0218759. doi: 10.1371/journal.pone.0218759 (PMC6705774; doi:10.1371/journal.pone.0218759)
Supplement: S1 Table — (DOCX) [file pone.0218759.s001.docx]

**S1 Table. Reasons for Not Initiating Treatment (Sample of Untreated Cohort With Mention of “DAA” in Patient Notes)**

| Reason, n (%) | **Patients Not Initiating DAA Treatment Within the 2-year Study Window**  **N = 287** |
| --- | --- |
| DAA therapy refused or declined | 249 (86.8) |
| Lost to follow-up from initial visit | 84 (29.3) |
| Insurance denied coverage of DAA or other cost barrier^a^ | 70 (24.4) |
| Physician decided not to treat patient with DAAs^b^ | 39 (13.6) |
| No mention of DAA treatment option in chart review | 36 (12.5) |
| Patient refused DAA treatment | 20 (7.0) |
|  |  |
| Patient received DAA therapy outside of the study period (in 2016) | 38 (13.2) |

DAA, direct-acting antiviral.

^a^Excludes absence of insurance.

^b^Due to disease state, drug use, alcohol use, etc. (i.e., physician made the decision, not the patient).
