# Supplementary material for: Initial uptake, time to treatment, and real-world effectiveness of all-oral direct-acting antivirals for hepatitis C virus infection in the United States: A retrospective cohort analysis
Source: PLoS One. 2019 Aug 22;14(8):e0218759. doi: 10.1371/journal.pone.0218759 (PMC6705774; doi:10.1371/journal.pone.0218759)
Supplement: S2 Table — (DOCX) [file pone.0218759.s002.docx]

**S2 Table. Baseline Characteristics and Demographics of the Direct-Acting Antiviral-Treated Cohorts Stratified
by Regimen (N = 830)**

| **Characteristic** | **Sofosbuvir/Ledipasvir N = 261** | **Sofosbuvir/Ledipasvir**  **+ Ribavirin**  **N = 121** | **Sofosbuvir +Ribavirin**  **N = 213** | **Sofosbuvir/Simeprevir**  **± Ribavirin**  **N = 187** | **PrOD**  **± Ribavirin N = 20** | **Others**  **N = 28** |
| --- | --- | --- | --- | --- | --- | --- |
| Age, mean (SD) | 57.2 (10.1) | 58.7 (8.1) | 55.8 (8.7) | 57.2 (9.1) | 55.1 (10.3) | 53.4 (10) |
| Sex, n (%) |  |  |  |  |  |  |
| Female | 115 (44.1) | 39 (32.2) | 75 (35.2) | 77 (41.2) | 6  (30) | 16  (57.1) |
| Race, n (%) |  |  |  |  |  |  |
| Black | 67 (26.1) | 21 (17.5) | 19 (9) | 42 (22.8) | 2 (10.5) | 1 (3.6) |
| Other | 2 (0.8) | 5 (4.2) | 3 (1.4) | 3 (1.6) |  |  |
| White | 188 (73.2) | 94 (78.3) | 189 (89.6) | 139 (75.5) | 17 (89.5) | 27 (96.4) |
| Missing, n | 4 | 1 | 2 | 3 | 1 | 0 |
| Ethnicity, n (%) |  |  |  |  |  |  |
| Hispanic or Latino | 1 (0.4) | 5 (4.1) | 6 (2.9) | 5 (2.7) | 1 (5.3) | 0 |
| Missing, n | 2 | 0 | 6 | 4 | 1 | 0 |
| Insurance, n (%) |  |  |  |  |  |  |
| Government | 18 (6.9) | 9 (7.4) | 11 (5.2) | 8 (4.3) | 1 (5) | 3 (10.7) |
| Medicaid | 35 (13.5) | 17 (14) | 40 (18.8) | 31 (16.6) | 2 (10) | 6 (21.4) |
| Medicare | 113 (43.5) | 43 (35.5) | 76 (35.7) | 75 (40.1) | 6 (30) | 10 (35.7) |
| Private | 87 (33.5) | 50 (41.3) | 75 (35.2) | 69 (36.9) | 11 (55) | 9 (32.1) |
| Self-Pay | 7 (2.7) | 2 (1.7) | 11 (5.2) | 4 (2.1) |  |  |
| Missing, n | 1 | 0 | 0 | 0 | 0 | 0 |
| Baseline ALT level mean (SD), (IU/L), | 54 (61.5) | 54.6 (45.7) | 56.4 (51.4) | 53.4 (43.5) | 48.7 (43.3) | 44.1 (43.8) |
| Missing, n | 44 | 22 | 36 | 25 | 5 | 5 |
| Anxiety, n (%) |  |  |  |  |  |  |
| Yes | 21 (8) | 9 (7.4) | 13 (6.1) | 11 (5.9) | 0 | 1 (3.6) |
| Baseline APRI level, mean (SD) | 1.1 (1.4) | 1.9 (1.8) | 1.6 (1.6) | 1.4 (1.3) | 1.6 (2) | 1.5 (1.6) |
| Missing, n | 47 | 25 | 43 | 31 | 6 | 6 |
| Baseline AST level, mean (SD), (IU/L) | 51.2 (38.3) | 61.1 (44.2) | 58.8 (46.3) | 56.4 (34) | 54.9 (50.2) | 51.4 (38.3) |
| Missing, n | 44 | 22 | 38 | 25 | 6 | 5 |
| Charlson Comorbidity Index, n (%) |  |  |  |  |  |  |
| 0 | 93 (35.6) | 29 (24) | 82 (38.5) | 69 (36.9) | 7 (35) | 14 (50) |
| 1 | 83 (31.8) | 47 (38.8) | 73 (34.3) | 55 (29.4) | 8 (40) | 6 (21.4) |
| 2 | 26 (10) | 11 (9.1) | 8 (3.8) | 12 (6.4) |  | 2 (7.1) |
| ≥3 | 59 (22.6) | 34 (28.1) | 50 (23.5) | 51 (27.3) | 5 (25) | 6 (21.4) |
| Cirrhosis, n (%) |  |  |  |  |  |  |
| Yes | 99 (37.9) | 65 (53.7) | 95 (44.6) | 87 (46.5) | 6 (30) | 9 (32.1) |
| CKD, n (%) |  |  |  |  |  |  |
| Yes | 22 (8.4) | 13 (10.7) | 6 (2.8) | 8 (4.3) | 3 (15) | 1 (3.6) |
| Decompensated cirrhosis, n (%) |  |  |  |  |  |  |
| Yes | 66 (25.3) | 47 (38.8) | 40 (18.8) | 32 (17.1) | 8 (40) | 7 (25) |
| Depression, n (%) |  |  |  |  |  |  |
| Yes | 23 (8.8) | 14 (11.6) | 13 (6.1) | 11 (5.9) | 1 (5) | 1 (3.6) |
| Diabetes, n (%) |  |  |  |  |  |  |
| Yes | 35 (13.4) | 14 (11.6) | 27 (12.7) | 24 (12.8) | 4 (20) | 1 (3.6) |
| Drug abuse, n (%) |  |  |  |  |  |  |
| Yes | 19 (7.3) | 12 (9.9) | 10 (4.7) | 4 (2.1) | 2 (10) | 1 (3.6) |
| FIB-4, n (%) |  |  |  |  |  |  |
| >3.5 | 73 (34.1) | 57 (59.4) | 89 (52.4) | 78 (50) | 6 (42.9) | 10 (45.5) |
| Missing, n | 47 | 25 | 43 | 31 | 6 | 6 |
| Fibrosis stage, n (%) |  |  |  |  |  |  |
| F0 | 15 (6.3) | 9 (7.9) | 12 (6.3) | 11 (6.1) | 2 (10.5) | 2 (7.4) |
| F1 | 41 (17.1) | 16 (14) | 29 (15.1) | 23 (12.8) | 6 (31.6) | 3 (11.1) |
| F2 | 37 (15.4) | 8 (7) | 16 (8.3) | 19 (10.6) | 1 (5.3) | 6 (22.2) |
| F3 | 33 (13.8) | 10 (8.8) | 20 (10.4) | 14 (7.8) | 1 (5.3) | 4 (14.8) |
| F4 | 114 (47.5) | 71 (62.3) | 115 (59.9) | 112 (62.6) | 9 (47.4) | 12 (44.4) |
| Missing, n | 21 | 7 | 21 | 8 | 1 | 1 |
| HCV genotype, n (%) |  |  |  |  |  |  |
| GT1 | 21 (8.6) | 3 (2.6) | 2 (1) | 24 (13) | 0 | 1 (3.7) |
| GT1a | 177 (72.5) | 77 (67) | 53 (26) | 120 (65.2) | 17 (89.5) | 1 (3.7) |
| GT1b | 44 (18) | 22 (19.1) | 11 (5.4) | 35 (19) | 1 (5.3) | 4 (14.8) |
| GT2 | 1 (0.4) | 2 (1.7) | 78 (38.2) | 5 (2.7) | 0 | 8 (29.6) |
| GT3 | 1 (0.4) | 4 (3.5) | 59 (28.9) | 0 | 1 | 12 (44.4) |
| GT4 | 21 (8.6) | 6 (5.2) | 1 (0.5) | 0 | 0 | 1 (3.7) |
| GT6 | 0 | 1 (0.9) | 0 | 0 | 0 | 0 |
| Missing, n | 17 | 6 | 9 | 3 | 1 | 1 |
| HBV infection, n (%) |  |  |  |  |  |  |
| Yes | 1 (0.4) | 2 (1.7) | 3 (1.4) | 2 (1.1) | 0 | 1 (3.6) |
| HIV infection, n (%) |  |  |  |  |  |  |
| Yes | 7 (2.7) | 1 (0.8) | 3 (1.4) | 1 (0.5) | 0 | 1 (3.6) |
| History of kidney transplant, n (%) |  |  |  |  |  |  |
| Yes | 5 (1.9) | 2 (1.7) | 1 (0.5) | 4 (2.1) | 2 (10) | 0 |
| History of liver  transplant, n (%) |  |  |  |  |  |  |
| Yes | 22 (8.4) | 37 (30.6) | 32 (15) | 20 (10.7) | 2 (10) | 4 (14.3) |
| Neutropenia, n (%) |  |  |  |  |  |  |
| Yes | 0 | 1 (0.8) | 2 (0.9) | 0 | 0 | 0 |
| Platelet count ≥100,000/µL, n (%) | 178 (79.8) | 53 (53.5) | 118 (66.3) | 101 (63.5) | 8 (57.1) | 10 (45.5) |
| Missing, n | 38 | 22 | 35 | 28 | 6 | 6 |
| Previous treatment  (interferon, pegylated interferon, ribavirin, first-generation DAA agent), n (%) |  |  |  |  |  |  |
| Yes | 44 (16.9) | 41 (33.9) | 78 (36.6) | 34 (18.2) | 4 (20) | 11(39.3) |
| Thrombo-cytopenia, n (%) |  |  |  |  |  |  |
| Yes | 6 (2.3) | 6 (5) | 3 (1.4) | 2 (1.1) | 2 (10) | 0 |
| ß-blocker use, n (%) |  |  |  |  |  |  |
| Yes | 101 (38.7) | 39 (32.2) | 84 (39.4) | 76 (40.6) | 7 (35) | 11 (39.3) |
| PPI use, n (%) |  |  |  |  |  |  |
| Yes | 94 (36) | 45 (37.2) | 84 (39.4) | 68 (36.4) | 5 (25) | 10 (35.7) |

ALT, alanine aminotransferase; APRI, aspartate aminotransferase to platelet ratio index; AST, aspartate aminotransferase; CKD, chronic kidney disease; DAA, direct-acting antiviral; FIB-4, Fibrosis 4; HBV, hepatitis B virus; HIV, human immunodeficiency virus; PPI, proton pump inhibitor.
