# Supplementary material for: Initial uptake, time to treatment, and real-world effectiveness of all-oral direct-acting antivirals for hepatitis C virus infection in the United States: A retrospective cohort analysis
Source: PLoS One. 2019 Aug 22;14(8):e0218759. doi: 10.1371/journal.pone.0218759 (PMC6705774; doi:10.1371/journal.pone.0218759)
Supplement: S3 Table — (DOCX) [file pone.0218759.s003.docx]

**S3 Table**. **Sustained Virologic Response Rates by Direct-Acting Antiviral Treatment Regimen in Participants With HCV
GT1–Infection (Evaluable Population)**

|  | **Regimen** | | | | | |
| --- | --- | --- | --- | --- | --- | --- |
| **Participants, n/N**  **SVR Rate (%)**  **(95% CI)** | **All** | **Sofosbuvir/Ledipasvir** | **Sofosbuvir/Ledipasvir +**  **Ribavirin** | **Sofosbuvir + Ribavirin** | **Sofosbuvir/Simeprevir ± Ribavirin** | **PrOD ± Ribavirin** |
| **All** | 355/423  83.92  (80.07–87.30) | 153/166  92.17  (86.98–95.76) | 55/65  84.62  (73.52–92.37) | 30/46  65.22  (49.75–78.65) | 105/132  79.55  (71.65– 86.07) | 9/10  90.00  (55.50– 99.75) |
| **Participants who completed treatment** |  |  |  |  |  |  |
| **8-week treatment (observed Tx)** | 15/18  83.33  (0.5858–0.9642) | 15/17  88.24  (63.56–98.54) | 0 | 0 | 0 | 0 |
| **12-week treatment** | 261/308  84.74  (0.8023– 0.8857) | 99/109  90.83  (83.77– 95.51) | 47/53  88.68  (76.97– 95.73) | 10/17  58.82  (32.92– 81.56) | 96/120  80.00  (71.72– 86.75) | 7/7  100.00  (59.04– 100.0) |
| **24-week treatment** | 68/82  82.93  (0.7302– 9034) | 38/39  97.44  (86.52– 99.94) | 7/11  63.64  (30.79– 89.07) | 16/24  66.67  (44.68– 84.37) | 1/1  100.00  (NA) | 1/1  100.00  (NA) |
| **Subgroups** |  |  |  |  |  |  |
| **Female** | 153/180  85.00  (78.93– 89.88) | 73/78  93.59  (85.67– 97.89) | 18/22  81.82  (59.72– 94.81) | 10/14  71.43  (41.90– 91.61) | 46/60  76.67  (64.96– 86.62) | 5/5  100.0  (47.82– 100.0) |
| **Male** | 202/243  83.13  (77.81– 87.61) | 8088  0.9091  (82.87– 95.99) | 37/43  86.05  (72.07– 94.70) | 20/32  62.50  (43.69– 78.90) | 59/72  81.94  (71.11– 90.02) | 4/5  80.00  (28.36– 99.49) |
| **Cirrhosis** | 154/197  78.17  (71.75– 83.73) | 56/63  88.89  (78.44– 95.41) | 22/25  88.00  (68.78–97.45) | 16/27  59.26  (38.80– 77.61) | 47/64  73.44  (0.6091– 0.8370) | 2/2  100.00  (47.82– 100.0) |
| **No cirrhosis** | 201/226  88.94  (84.10– 92.71) | 97/103  94.17  (87.75– 97.83) | 33/40  82.50  (67.22– 92.66) | 14/19  73.68  (48.80– 90.85) | 58/68  85.29  (74.61– 92.72) | 7/8  87.50  (47.35– 99.68) |
| **Black** | 82/89  92.13  (84.46– 96.78) | 43/44  97.73  (87.98– 99.94) | 9/9  100.0  (66.37– 100.0) | 7/9  77.78  (39.99– 97.19) | 22/25  88.00  (68.78– 97.45) | 1/2  50.00  (1.26– 98.74) |
| **Non-black** | 273/334  81.74  (77.17– 85.73) | 110/122  90.16  (83.45– 94.81) | 46/56  82.14  (69.60– 91.09) | 23/37  62.16  (44.76– 77.54) | 83/107  77.57  (68.49– 85.07) | 8/8  100.0  (63.06– 100.0) |
| **Treatment- experienced** | 104/126  82.54  (74.77– 88.72) | 35/35  100.0  (90.00– 100.0) | 23/27  85.19  (66.27– 95.81) | 19/30  63.33  (43.86– 80.07) | 23/29  79.31  (60.28– 92.01) | 3/3  100.0  (29.24– 100.0) |
| **Treatment-naive** | 251/297  84.51  (79.89– 88.43) | 118/131  90.08  (83.63– 94.61) | 32/38  84.21  (68.75– 93.98) | 11/16  68.75  (41.34– 88.98) | 82/103  79.61  (70.54– 86.91) | 6/7  85.71  (42.13– 99.64) |
| **Platelet count ≥100,000/µL** | 220/248  88.71  (84.10– 92.37) | 112/121  92.56  (86.35– 96.54) | 25/27  92.59  (75.71– 99.09) | 19/25  76.00  (54.87– 90.64) | 60/70  85.71  (75.29– 92.93) | 3/4  75.00  (19.41– 99.37) |
| **Platelet count <100,000/µL** | 86/115  74.78  (65.83–82.42) | 23/24  95.83  (78.88– 99.89) | 22/27  81.48  (61.92– 93.70) | 8/16  50.00  (24.65– 75.35) | 29/43  67.44  (51.46– 80.92) | 3/3  100.0  (29.24– 100.0) |

CI, confidence interval; GT, genotype; HCV, hepatitis C virus; PrOD, paritaprevir/ritonavir/ombitasvir/dasabuvir.
