# Supplementary figures and images for: Initial uptake, time to treatment, and real-world effectiveness of all-oral direct-acting antivirals for hepatitis C virus infection in the United States: A retrospective cohort analysis
Source: PLoS One. 2019 Aug 22;14(8):e0218759. doi: 10.1371/journal.pone.0218759 (PMC6705774; doi:10.1371/journal.pone.0218759)

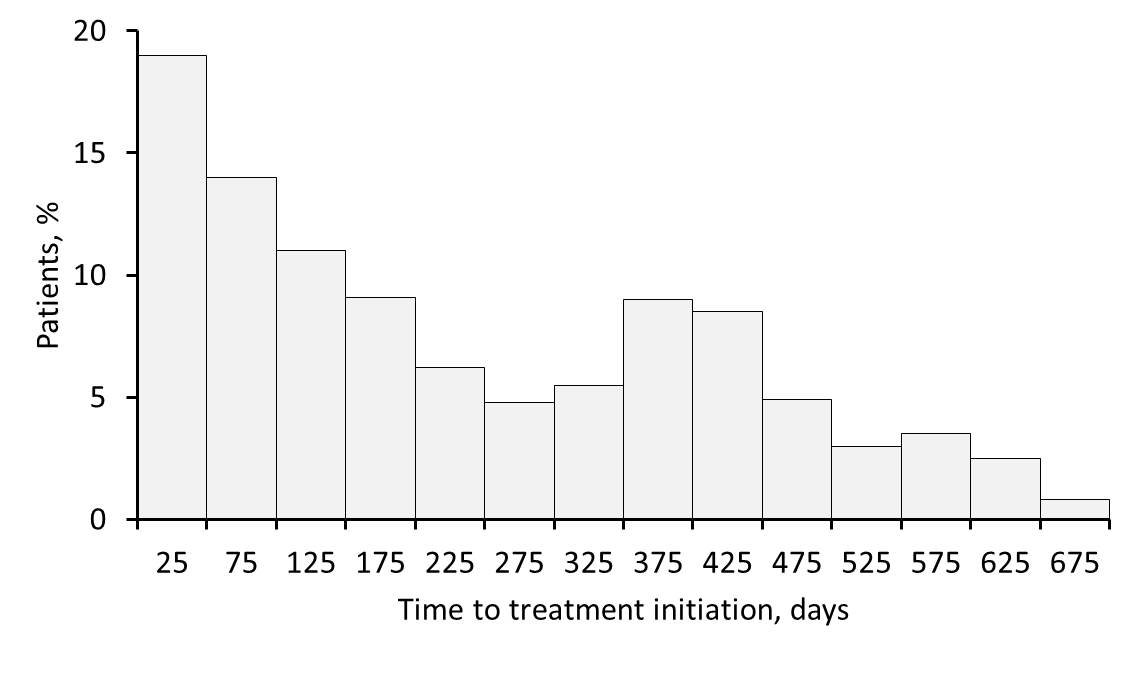


**Figure S1.** Histogram of time to DAA treatment initiation.

Supplement: S1 Fig — (DOCX) [file pone.0218759.s004.docx]
